# Assessment, classification and treatment of calcinosis as a complication of juvenile dermatomyositis: a survey of pediatric rheumatologists by the childhood arthritis and rheumatology research alliance (CARRA)

**DOI:** 10.1186/s12969-017-0199-4

**Published:** 2017-09-21

**Authors:** A. B. Orandi, K. W. Baszis, V. R. Dharnidharka, A. M. Huber, M. F. Hoeltzel, for the CARRA Juvenile Myositis subgroup

**Affiliations:** 1 Department of Pediatrics, Division of Rheumatology, Washington University School of Medicine, 660 S. Euclid Ave, Campus Bo 8116, St. Louis, Missouri 63110 USA; 2 Department of Pediatrics, Division of Nephrology, Washington University School of Medicine, St. Louis, Missouri USA; 3 Department of Pediatrics, Division of Rheumatology, IWK Health Centre and Dalhousie University, Halifax, Nova Scotia Canada; 4 Department of Pediatrics, Division of Rheumatology, University of Michigan Medical School, Ann Arbor, Michigan USA

**Keywords:** Juvenile dermatomyositis, Calcinosis, Survey, CARRA, Treatment

## Abstract

**Background:**

There is no standardized approach to the management of JDM-associated calcinosis and its phenotypes. Current knowledge of treatment outcomes is confined to small series and case reports. We describe physician perspectives toward diagnostic approach, classification and treatment directly targeting calcinosis, independent of overall JDM therapy.

**Methods:**

An electronic survey of 22 questions was organized into sections regarding individual practices of assessment, classification and treatment of calcinosis, including perceived successes of therapies. Invitations to complete the survey voluntarily and anonymously were sent to CARRA physician members and the Pediatric Rheumatology Bulletin Board, an electronic list-serv. Results were analyzed by descriptive statistics and chi-square analyses.

**Results:**

Of 139 survey responses, 118 were included in analysis. Of these, 70% were based in the USA and 88 (75%) were CARRA members. Only 17% of responders have seen more than 20 cases of calcinosis, and only 28% perform screening imaging studies on new JDM diagnoses. Increasing systemic immunosuppression is first-line therapy for 67% of respondents. Targeted therapy against calcinosis is most often instituted for symptomatic patients. IVIG and bisphosphonates are most frequently used and considered most successful, but many other agents are used. Experienced physicians are more likely to use bisphosphonates, calcium channel blockers and topical sodium thiosulfate (*p*< 0.002 or lower).

**Conclusions:**

Coexisting JDM disease activity influences whether calcinosis is considered active disease or targeted directly. Experience treating JDM-related calcinosis is low, as are rates of formal screening for calcinosis. Experienced physicians are more likely to use non-immunosuppressive treatments.

**Electronic supplementary material:**

The online version of this article (doi: 10.1186/s12969-017-0199-4) contains supplementary material, which is available to authorized users.

## Background

Juvenile dermatomyositis (JDM) is the most common inflammatory myopathy of childhood, though still rare, with an estimated incidence of 3.2 children per million per year [1]. The cardinal features include proximal muscle weakness and characteristic rash, but multiple other organs can be involved and contribute to morbidity and mortality, including the gastrointestinal, cardiac and pulmonary systems. With improved disease understanding and treatments, mortality has been significantly reduced [2, 3], but long-term morbidity and treatment toxicity remain as challenges [4]. A prominent contributor to overall morbidity is calcinosis, the accumulation of dystrophic carbonate apatite [5] in skin and soft tissues, both a trademark and feared complication whose presence has been unyielding in the face of other advances. Its staying power is owed to an incomplete understanding of both its pathogenesis and actionable risk factors that could support alterations in management [6–8]. Although it is reported that early aggressive treatment can prevent the development of calcinosis [9–11], the incidence continues to remain at an estimated 30–40% of patients through various cohort and registry studies [12–15].

To date, there are no randomized controlled trials or standardized recommendations for the treatment of calcinosis as it occurs in JDM. Many therapeutic agents with different mechanisms of action have been used, with none showing a consistently positive response. In a recent review, nearly 20 different therapies have been described to have positive responses in published cases [15]. Further complicating the management of this condition are the varying phenotypes [16, 17], such as circumscripta, tumoral, universalis and exoskeleton lesions, as well as the inconsistent timing of calcinosis respective to other disease features [18–20]. While the ultimate goal is to improve outcomes of patients with calcinosis, our aim is to describe the current practices of pediatric rheumatologists, including approaches to assessment, classification and treatment, thereby consolidating consensus opinions that can be formally studied. This technique has been successfully implemented in the overall treatment of JDM by the Childhood Arthritis and Rheumatology Research Alliance (CARRA), whose consensus treatment plans are currently being instituted and analyzed [21–24].

## Methods

Using the REDCap platform, an electronic survey was created, consisting of 22 questions divided into four sections: demographic characteristics, assessment, classification and treatment (Additional file 1). For phenotype classification questions, calcinosis phenotypes were described as follows: Circumscripta; superficial plaques or nodules. Tumoral; larger nodules that extend into deeper layers. Universalis; involvement along fascial planes of muscles or tendons. Exoskeleton; hardened deposits over a surface area. For treatment choice-related questions, ‘immunomodulatory’ agents are those which directly suppress or modulate the immune system whereas ‘alternative’ agents are those with non-immunosuppressive actions, such as altering calcium and/or phosphorous metabolism. Any treatment success-related questions are defined as physician perceived success. For questions related to situational treatment, ‘targeted treatment’ refers to a treatment chosen for calcinosis specifically and not any other co-existing JDM disease activity.

Physicians, including fellows-in-training, who specialize in pediatric, adult, combined rheumatology or immunology were invited by e-mail to complete the survey. Participants were asked to respond according to their personal experience, not that of institution or group practices or based on the medical literature. Respondents were asked to quantify their experience by years of practice and lifetime cases seen of JDM-associated calcinosis. In January 2016, the survey was electronically sent to 303 voting and trainee physician members of CARRA; a North American collaborative research organization and to the Pediatric Rheumatology Bulletin Board, an international electronic list-serv. Members of CARRA who are also on the Bulletin Board were asked to only respond once. All respondents voluntarily and anonymously completed the survey.

Physicians of different experience levels, determined by years of practice and by number of cases seen, were captured, and the discrepancy between experience levels highlighted an important finding: because of the rare incidence of JDM, even some seasoned physicians had seen few cases of calcinosis. Because of this finding, the number of cases seen was used as the metric of experience instead of years in practice. Therefore, a physician who is experienced with treating JDM-associated calcinosis was defined as having seen greater than 10 cases. The results of the survey were analyzed by descriptive statistics and chi-square analyses for comparisons between physicians who have seen greater and less than 10 cases. Analysis was performed using IBM SPSS Statistics (version 24), Armonk, NY.

## Results

**Fig. 1:**
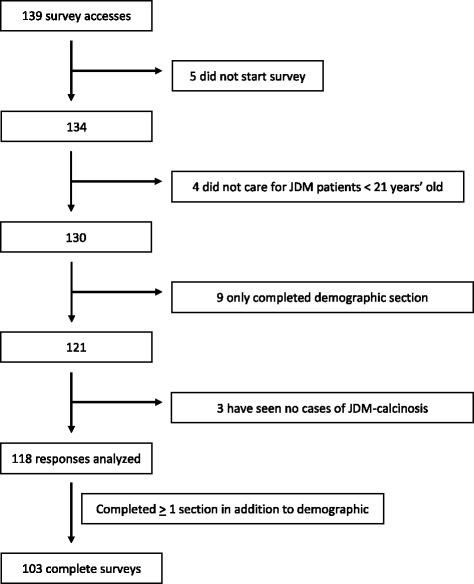
Number of survey respondents

A total of 139 individuals accessed the survey with 5 individuals excluded for not answering any questions. Four respondents were excluded for no personal experience with JDM patients less than 21 years of age, and 3 were excluded for no personal experience treating JDM-associated calcinosis. An additional 9 were excluded for only completing the demographic section. A total of 118 respondents completed at least one full survey section in addition to demographic characteristics, qualifying for analysis, with a total of 103 respondents completing the survey entirely (Fig. 1). CARRA members constituted 75% (88/118) of the analyzed responses and based on the number of CARRA members who received the survey invitation the CARRA response rate is estimated at 30% (88/303).

Less than 5% of respondents practiced something other than pediatric rheumatology. Approximately 70% of respondents were based in the United States, with Europe the next most common location at 14%. The distribution of experience by years showed nearly equal young providers (current fellow to 5 years) and those with more than 20 years experience, at 27% for both, with lesser numbers in between. When experience was measured by number of cases seen, the majority, 62%, have seen between 1 and 10 cases, with only 17% having seen greater than 20 cases. Of these who have seen more than 20 cases, 16 (80%) have more than 15 years experience. However, of those with more than 15 years experience, 39% had seen less than 10 cases. Complete respondent characteristics are detailed in Table 1.

**Table 1:** Respondent characteristics

Characteristic	n (%)
Practice scope	
Pediatric Rheumatology	114 (96.6)
Adult Rheumatology	1 (0.8)
Combined adult/pediatric Rheumatology	3 (2.5)
Immunology	0 (0)
Practice location	
United States	82 (69.5)
Canada	8 (6.8)
Central/South America	9 (7.6)
Europe	16 (13.6)
Asia/India	1 (0.8)
Other (Turkey, Kenya)	1, 1 (0.8)
CARRA Member	
Yes	88 (74.6)
No	30 (25.4)
Experience (by years of practice)	
Current fellow to 5 years	32 (27.1)
6 to 10 years	30 (25.4)
11 to 15 years	10 (8.5)
16 to 20 years	14 (11.9)
More than 20 years	32 (27.1)
Experience (by # of JDM-calcinosis cases seen)	
1 to 10 cases	74 (62.7)
11 to 20 cases	24 (20.3)
21 to 50 cases	18 (15.3)
More than 50	2 (1.7)

Demographic characteristics of all survey respondents included in analysis

### Assessment

When evaluating a new JDM diagnosis, 60% use only history and/or physical exam to screen for calcinosis. If history and physical exam are negative, 33 (28%) perform imaging studies. Only 11% perform no formal assessment. For presence or suspicion of calcinosis, radiographs are the initial imaging studies for 88%, whereas 18% use ultrasound, 17% MRI and 4% computed tomography. If calcinosis is suspected or found, nearly equal numbers send laboratory studies evaluating calcium and vitamin D levels or no labs at all (45% each). Once treatment is initiated, 95% use physical exam to monitor response to therapy (if phenotype is able to be examined), whereas 70% use imaging. Of these, plain radiographs are used by 72% of respondents, 15% use MRI, 7% use ultrasound and 4% use computed tomography.

### Classification–Calcinosis phenotype

Two-thirds of respondents believed it was important to classify the phenotype of calcinosis lesions, with selected rationale of the phenotype affecting treatment type (surgical or medical), treatment aggression or predicting response to treatment. Others listed different expected morbidity or severity from specific phenotypes. Opponents of classification believed that only the presence of calcinosis was important, regardless of phenotype. When asked to rate phenotypes by perceived severity or worse prognosis, 65% listed universalis, followed by exoskeleton lesions at 57% and tumoral at 42%.

### Classification–Disease activity

When asked to characterize “active JDM disease”, nearly all respondents reported the presence of skin and muscle disease (defined as presence of rash, nail fold capillary changes, muscle weakness, elevation of muscle enzymes, abnormal muscle imaging or EMG findings) as active JDM disease, opinions that were unchanged by the presence of new or persistent/refractory calcinosis. However, in the absence of skin and muscle disease, 73% reported new onset calcinosis as active JDM disease, while only 14% regarded absent skin and muscle disease with persistent/refractory calcinosis as active JDM disease. Given the same scenarios of JDM disease states, respondents were asked to consider *targeted* treatment of calcinosis, independent of therapy prescribed for overall JDM disease activity. The rates of consideration for targeted treatment were higher in scenarios where skin and muscle disease activity were decreased and persistence of calcinosis increased, such that only 56% would consider targeted treatment of calcinosis for active skin and muscle disease with new calcinosis, but 80% would consider targeted treatment of calcinosis for absent skin and muscle disease with persistent/refractory calcinosis. Only 43% would consider targeted treatment of calcinosis in all scenarios, and 9% would not consider targeted therapy in any scenario.

The features of calcinosis lesions that increase the likelihood of targeted therapy, independent of overall JDM therapy, are more likely to be symptomatic features as follows: Functional impairment and pain were listed by 97% of respondents, whereas threat to adjacent organs and recurrent infections were listed by 89 and 80%, respectively. Cosmesis (or psychosocial impact) was listed by 71%. Only 29% listed calcinosis phenotype, and 8% listed presence of certain myositis antibodies (listed as Jo-1, MDA-5 and NXP2) that would increase the likelihood of targeted treatment. When these features were ranked in order of importance to considering targeted treatment, functional impairment was ranked highest by 41%, pain ranked second by 40%, and location (as threatening to other organs) third at 37%.

### Treatment–Surgical

As first line therapy, only 5% of respondents would refer for surgical excision. However, if the type of lesion is amenable to surgical removal, 24% believed every such case should have a surgical evaluation. Further, 60% would consider surgery if the lesion(s) caused significant limitation in mobility or significant pain, but 38% believe it should be used only if medical therapy failed. Only 4% believe there is no role for surgery in the management of calcinosis.

### Treatment–Medical

**Fig. 2:**
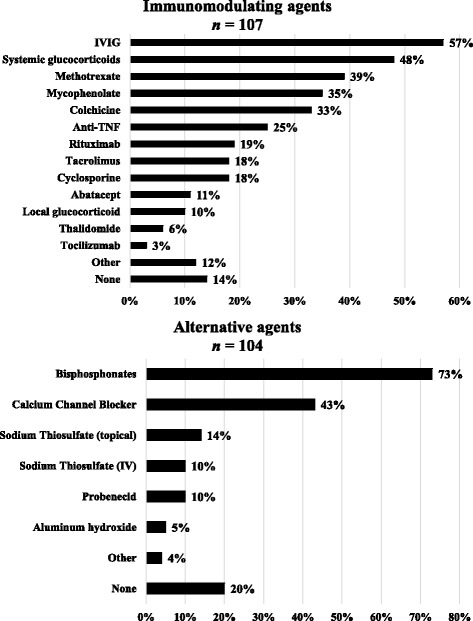
Treatment categories and frequency of use among all respondents

Therapies were separated into two categories: immunomodulatory and alternative agents, the latter including drugs with non-immunosuppressive actions, such as altering calcium and phosphorus metabolism. The drugs in each category and their frequencies of use are displayed in Fig. 2. For the patient developing or presenting with calcinosis, increasing or adding systemic immunosuppression was first line therapy for 67% of respondents, followed by alternative agents at 13%. A total of 14 (13%) would prescribe no specific treatment other than ‘standard’ therapy for overall JDM activity.

Of the immunomodulatory agents listed, the most frequently used include IV immune globulin (IVIG) by 61 (57%), systemic glucocorticoids by 48% and methotrexate (specifically for calcinosis) at 40%. Less frequently used agents include TNF-alpha inhibitors at 25%, rituximab at 19% and abatacept at 11%. Other immunomodulatory agents that were not listed, but totaled 12%, included anakinra, cyclophosphamide, lenalinomide and sirolimus.

When asked to rank which treatment was perceived to be most successful against calcinosis, IVIG was ranked first by 30% of users and second by 26% of users, whereas systemic glucocorticoids were ranked first by 32% of users and second by 17%. Methotrexate was ranked first by 24% and second by 9% of respondents. Only mycophenolate, tacrolimus, colchicine and cyclosporine were ranked in the top three by greater than 10% of respondents; they were listed second by 20, 12, 12%, and third by 10% respectively.

For alternative agents, bisphosphonates were the most frequently used (73% of respondents), followed by calcium channel blockers at 43%. Intravenous sodium thiosulfate was used by 10%, with 14% using the topical formulation. Other agents not listed that were used include warfarin and minocycline (totaling 4%). Of note, 20% of respondents have not used any of the alternative agents.

When ranked according to perceived success against calcinosis, approximately 60% ranked bisphosphonates most effective, whereas only 15% ranked calcium channel blockers highest, with an additional 30% ranking second most effective. Although few respondents have used intravenous sodium thiosulfate, it was perceived as most effective by three individuals (30%).

### Experience differences

**Fig. 3:**
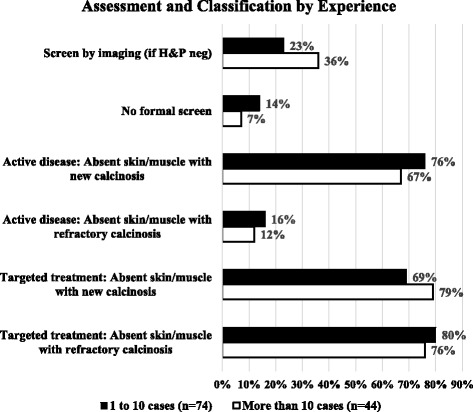
Assessment and classification by experience in regards to using imaging to screen for calcinosis if none is apparent by history or physical exam or if no formal screen is done. Respondent opinion of what constitutes active disease based on the presence or absence of skin/muscle disease with or without new or refractory calcinosis; and if they would consider targeted treatment against calcinosis irrespective of other disease activity in the same scenarios

Separating experience by those who have seen more or less than 10 cases, there was no statistically significant difference regarding certain assessment and classification practices (Fig. 3). Roughly equal numbers screen new diagnoses with imaging or have no formal screening process. Neither group was more or less likely to consider new or stable calcinosis as active JDM disease in the absence of skin and muscle disease. Similarly, there was no difference in the consideration to target treatment against calcinosis in the absence of skin and muscle activity.

**Fig. 4:**
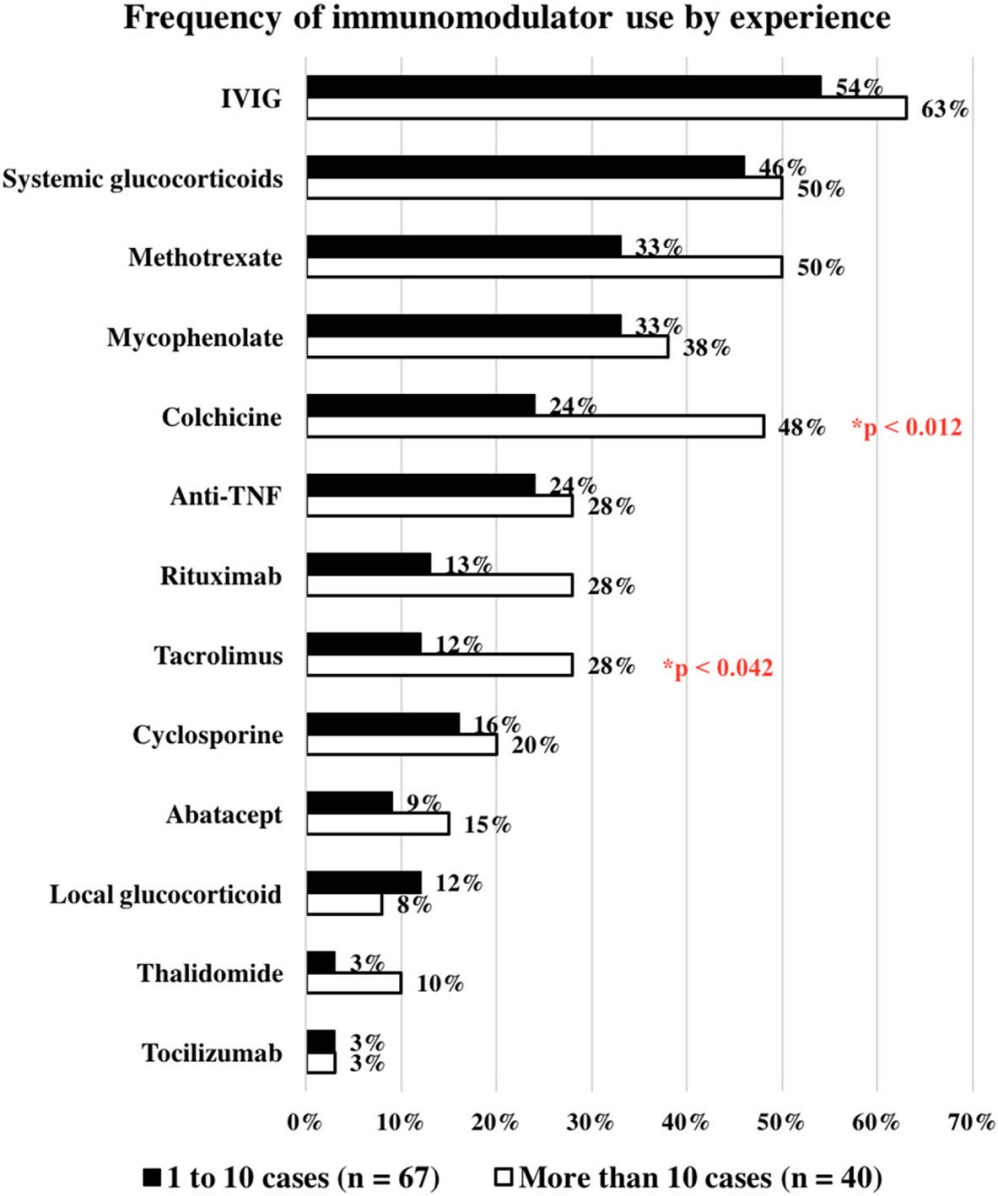
Frequency of immunomodulatory use by experience

**Fig. 5:**
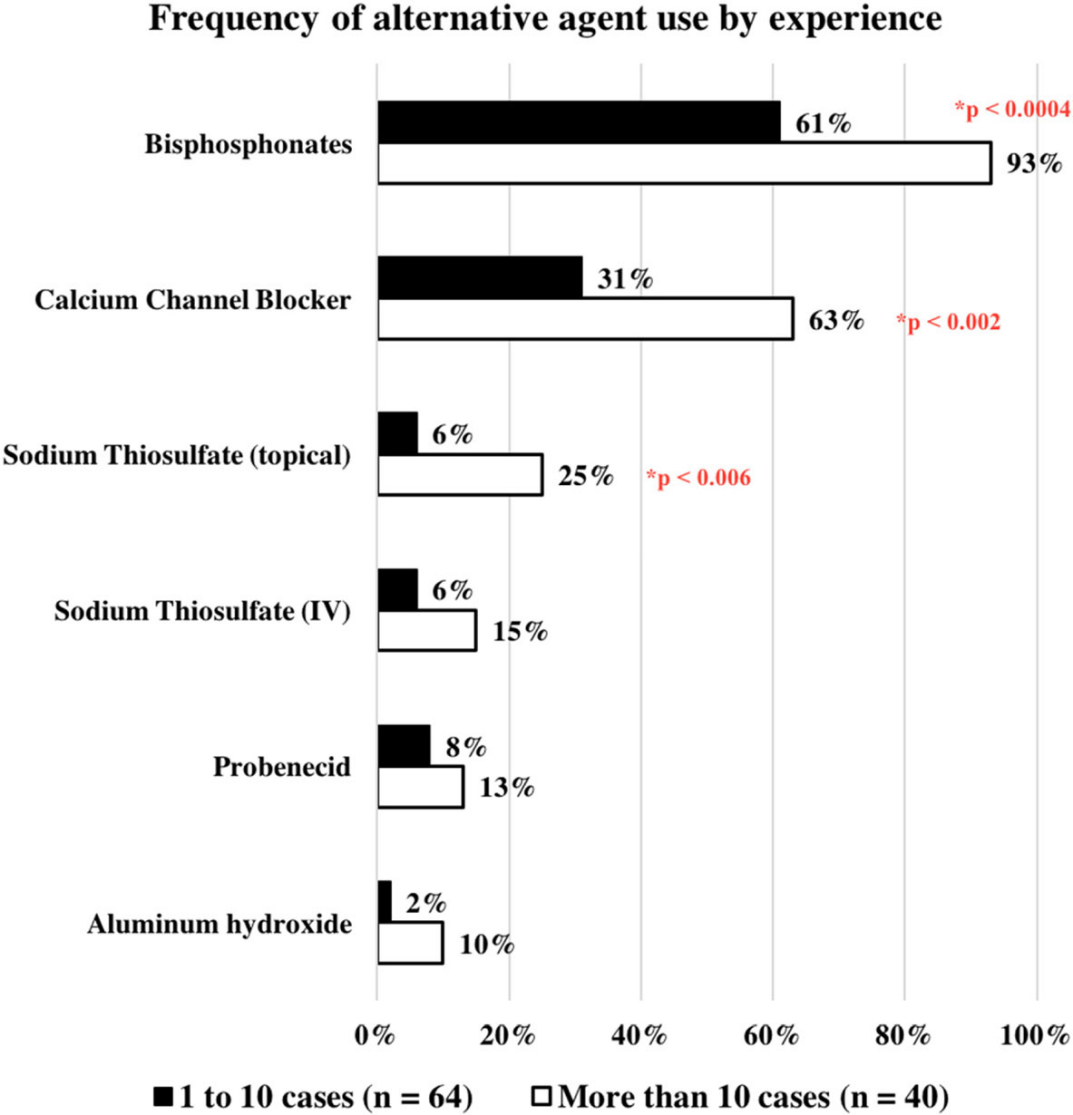
Frequency of alternative agent use by experience

Examining the differences in treatment use, both groups reported highest use of the same top three immunomodulatory agents: IVIG, systemic glucocorticoids and methotrexate (Fig. 4). For every agent except local glucocorticoids, the more experienced respondents reported greater frequency of use, but only colchicine (19/40 versus 16/67, Pearson chi-square value = 6.348, *p* = 0.012) and tacrolimus reached statistical significance (11/40 versus 8/67, Pearson chi-square value = 4.152, *p* = 0.042). Regarding alternative medications, again each group had the same top two most frequently used agents: bisphosphonates and calcium channel blockers (Fig. 5). However, the increased use by more experienced respondents reached statistical significance for bisphosphonates (37/40 versus 39/64, Pearson chi-square value = 12.464, *p*< 0.001), calcium channel blockers (25/40 versus 20/64, Pearson chi-square value = 9.793, *p* = 0.002) and topical sodium thiosulfate (10/30 versus 4/60, Pearson chi-square value = 7.429, *p* = 0.006).

## Discussion

Calcinosis remains a significant source of morbidity for many JDM patients, yet it is poorly understood and lacks uniform treatment approaches compared to other aspects of JDM. This survey describes the approach and management of calcinosis by a large number of pediatric rheumatologists.

The inconsistency in the published literature regarding therapeutic effectiveness can be explained by the relative inexperience of physicians and the multitude of different treatments and treatment scenarios. To date there have been no published reports comparing therapeutic effectiveness against calcinosis when there is active versus absent skin and muscle disease. Additionally, not all published reports list failed therapies prior to success or concurrent background medications [25]. Part of the discrepancy between number of cases seen and experience is owed to practice type (size, university affiliation) and location (city, region, competing practices), which was not assessed in this survey. However, another potential contributor is the low percentage of physicians who routinely screen for calcinosis beyond patient history and physical examination. Based on our results, only 28% perform additional imaging if history and exam are negative for calcinosis while 11% perform no formal screening. This low frequency may be in part due to the lack of any studied screening methods. It is well-reported that lesions can be present at diagnosis or much later [26]. Deeper lesions such as exoskeleton and universalis phenotypes may not be apparent until a critical size or mass effect is reached. A lack of screening is unique to calcinosis when compared to other JDM complications, such as interstitial lung disease, where screening with pulmonary function testing and/or CT imaging is routinely performed since it is known that even asymptomatic children can have long-term sequelae [27–29].

Also revealing was the expressed understanding of the role calcinosis plays in active disease and the decision to treat with therapies targeted at calcinosis. It was clear that respondents believed the new development of calcinosis with otherwise absent skin and muscle disease represents active disease (73%), however, only 56% would consider targeted treatment of calcinosis in the most agreed upon active systemic disease state. Combining these results provides stark evidence that the majority (80% combined) prefer to prescribe either systemic immunosuppression or a regimen based on overall JDM activity with only symptomatic complaints prompting targeted treatment against calcinosis. Importantly, none of the above showed significant differences among physicians of different experience levels. The results showed, however, that experienced physicians may be more willing to use unconventional therapies in the treatment of this complication. Colchicine and tacrolimus were two immunomodulatory agents used significantly more by experienced physicians for treatment of calcinosis, but they are not mainstays of immunosuppressive treatment of JDM as a whole. Similarly, although both groups used alternative agents, experienced physicians were significantly more likely to use bisphosphonates, calcium channel blockers and topical sodium thiosulfate.

Our study has several limitations, including the relatively low response rate for CARRA members. In addition, the majority of respondents were from North America and/or CARRA members. Although our intent to include the pediatric rheumatology bulletin board community was to obtain a broad scope of practice across the field, the results cannot be reliably generalized to the field at-large and perhaps more accurately represent North American and CARRA members with an interest in JDM. It is possible that many chose not to complete the survey due to a lack of experience with JDM-associated calcinosis. Thus, an increase number of respondents with only a few cases seen may not significantly alter the results. Another limitation of our survey (and others that attempt to describe practice), is that physicians may answer based on information other than personal experience and duplicate their responses despite instructions to discourage these types of responses. Many of the questions were based on hypothetical situations, without complete details that would be present in case-based surveys. This survey did not directly address the successes of each physician’s practices, complications of treatment or cost-effectiveness. Finally, this study is not intended to recommend specific treatment(s) for JDM-associated calcinosis but to describe the current trends in management and highlight the need for increased evidence in assessment and treatment efficacy.

## Conclusion

Calcinosis is considered a major source of morbidity, but often excluded as a metric of clinically active or inactive disease [30], leaving many physicians with limited experience. There is a lack of formal screening, and most direct treatment decisions are based on symptomatic complaints, which is at odds with the moderate numbers who consider it a component of active disease. Increased formal research is needed, which could include screening methods, consensus treatment plans that incorporate disease activity, and the study of combination therapies in different patient subgroups.

## Additional file

Additional file 1: JDM Calcinosis Survey. (PDF 63 kb)

## Abbreviations

CARRA: Childhood arthritis and rheumatology research alliance
IVIG: Intravenous immune globulin
JDM: Juvenile dermatomyositis.
