# Supplementary material for: Assessment, classification and treatment of calcinosis as a complication of juvenile dermatomyositis: a survey of pediatric rheumatologists by the childhood arthritis and rheumatology research alliance (CARRA)
Source: Pediatr Rheumatol Online J. 2017 Sep 21;15:71. doi: 10.1186/s12969-017-0199-4 (PMC5609060; doi:10.1186/s12969-017-0199-4)
Supplement: Additional file 1: — JDM Calcinosis Survey. (PDF 63 kb) [file 12969_2017_199_MOESM1_ESM.pdf]

JDM Calcinosis Survey

The Juvenile Dermatomyositis (JDM) Calcinosis subcommittee of the Childhood Arthritis and Rheumatology Research Alliance (CARRA) is surveying Pediatric Rheumatologists for their approach to assessing and treating calcinosis as a complication of JDM.

Please note: Responses should pertain to treating calcinosis associated ONLY with JDM, and NOT with other diseases associated with calcinosis, such as scleroderma, mixed connective tissue disease, overlap syndromes, etc.

Questions will be organized by demographics, assessment, classification and treatment.

To avoid overlapping responses, please only refer to patients who you have cared for directly, as opposed to patients who may be cared for by colleagues.

Demographics

Do you provide clinical care to patients with JDM, age 21 years and younger?

- ◯ Yes
- ◯ No

What most accurately describes your scope of practice?

- ◯ Pediatric rheumatology.
- ◯ Adult rheumatology.
- ◯ Combined adult/pediatric rheumatology.
- ◯ Immunology.
- ◯ Other

State "other" which most accurately describes your scope of practice:

____

What location best describes your practice?

- ◯ USA
- ◯ Canada
- ◯ Central/South America
- ◯ Europe
- ◯ Asia/India
- ◯ Other

State 'other' location of your practice:

____

Are you a member of the Childhood Arthritis and Rheumatology Research Alliance (CARRA)?

◯ Yes
◯ No

How long have you practiced in your field since completing subspecialty training?

◯ current fellow to 5 years
◯ 6 to 10 years
◯ 11 to 15 years
◯ 16 to 20 years
◯ More than 20 years

How many JDM patients with calcinosis have you directly cared for?

◯ None
◯ 1 to 10
◯ 11 to 20
◯ 21 to 50
◯ More than 50

Assessment

With a new JDM diagnosis, what is your usual method of assessing (screening) for calcinosis?
(SELECT ALL THAT APPLY)

☐ History/patient report symptoms.
☐ Physical exam.
☐ Laboratory studies.
☐ Imaging (when calcinosis is not evident by history or physical exam)
☐ No formal assessment or screen at diagnosis.
☐ Other

State 'other' screening method:

____

If calcinosis is suspected or found, what initial imaging studies, if any, do you perform?
(SELECT ALL THAT APPLY)

☐ Radiograph (X-ray)
☐ Ultrasound (US)
☐ Magnetic resonance imaging (MRI)
☐ Computed tomography (CT)
☐ Scintigraphy (PET or other nuclear medicine scan)
☐ None
☐ Other

State 'other' imaging modality:

____

If calcinosis is suspected or found, what specific laboratory studies, if any, do you obtain?
(SELECT ALL THAT APPLY)

☐ Total calcium with albumin or ionized calcium
☐ Parathyroid hormone
☐ Vitamin D level
☐ Urinary calcium levels
☐ None
☐ Other

State 'other' laboratory studies:

____

Classification

Which of the following scenarios do you consider 'active JDM disease'?

Please note: skin and muscle disease includes rash, nail fold capillary changes, muscle weakness, elevation of muscle enzymes, abnormal imaging or EMG studies
(SELECT ALL THAT APPLY)

☐ Active skin and/or muscle disease with new calcinosis.
☐ Active skin and/or muscle disease with persistent or refractory calcinosis.
☐ Absent skin and/or muscle disease with new calcinosis.
☐ Absent skin and/or muscle disease with persistent or refractory calcinosis.
☐ None of these (ie other - please explain)

State 'other' scenarios you consider active disease that involves calcinosis:

In which of the following scenarios would you consider targeted treatment of calcinosis, independent of treatment for overall disease activity?

Please note: skin and muscle disease includes rash, nail fold capillary changes, muscle weakness, elevation of muscle enzymes, abnormal imaging or EMG studies
(SELECT ALL THAT APPLY)

☐ Active skin and/or muscle disease with new calcinosis.
☐ Active skin and/or muscle disease with persistent or refractory calcinosis.
☐ Absent skin and/or muscle disease with new calcinosis.
☐ Absent skin and/or muscle disease with persistent or refractory calcinosis.
☐ None of these (ie other - please explain)

State 'other' scenarios that you would consider targeted treatment of calcinosis:

Which features increase the chance you will prescribe adjunctive therapy targeted against calcinosis, independent of therapy for the given overall disease state?
(SELECT ALL THAT APPLY)

☐ A specific calcinosis phenotype
☐ Functional impairment (affecting mobility or range of motion).
☐ Significant pain or discomfort.
☐ Threat to adjacent organs (i.e. location).
☐ Recurrent infections.
☐ Presence of certain myositis antibodies
☐ Specific genotype
☐ Other signs of active disease (skin, muscle or both)
☐ Cosmesis (psychosocial impact)
☐ None.
☐ Other

State which 'myositis antibodies' increase the chance you will prescribe targeted adjunctive therapy against calcinosis:

____

State which 'genotype' increases the chance you will prescribe targeted adjunctive therapy against calcinosis:

____

State 'other' features not listed, that increase the chance you will prescribe adjunctive therapy targeted against calcinosis:

____

Rank the five features (in order of importance) that increase the chance you will prescribe adjunctive therapy targeted against calcinosis, independent of therapy for the given overall disease state.

	First	Second	Third	Fourth	Fifth
A specific calcinosis phenotype	◯	◯	◯	◯	◯
Functional impairment (affecting mobility or range of motion)	◯	◯	◯	◯	◯
Significant pain or discomfort	◯	◯	◯	◯	◯
Threat to adjacent organs (i.e. location)	◯	◯	◯	◯	◯
Recurrent infections	◯	◯	◯	◯	◯
Presence of certain myositis antibodies	◯	◯	◯	◯	◯
Specific genotype	◯	◯	◯	◯	◯
Other signs of active disease (skin, muscle or both)	◯	◯	◯	◯	◯
Cosmesis (psychosocial impact)	◯	◯	◯	◯	◯

When calcinosis is present, do you feel it is important to classify by phenotype (i.e., superficial plaques, large nodules, exoskeleton, etc)?

◯ Yes
◯ No

State your reasons for classifying or not classifying calcinosis phenotype

When present, which phenotype of calcinosis do you consider more severe or portends a worse prognosis? (SELECT ALL THAT APPLY)

☐ Calcinosis circumscripta (superficial plaques or nodules).
☐ Calcinosis "tumoral" (larger nodules that extend into deeper layers).
☐ Calcinosis universalis (along fascial planes of muscles or tendons).
☐ Exoskeleton Calcinosis (hard deposits over a surface area).
☐ Any type with or without active disease.
☐ None (ie phenotype is not predictive of prognosis.)
☐ Other

State which 'other' phenotype do you consider more severe or has a worse prognosis:

____

Treatment

In general, what is your first line treatment for the patient developing or presenting with calcinosis?

◯ Referral for surgical excision (if type is amenable to surgery).
◯ Increase or Add systemic immunosuppression.
◯ Start local immunosuppression (topical or injectable).
◯ Prescribe drugs that alter calcium or phosphate metabolism.
◯ None other than 'standard' treatment for given disease activity.
◯ Other

State 'other' first line treatment for the patient presenting with or developing calcinosis:

____

Do you believe in a role for surgical excision of calcinosis as a complication of JDM?
(SELECT ALL THAT APPLY)

☐ Yes if type is amenable to surgery, every case should be evaluated by a surgeon.
☐ Yes but only if causing significant limitation in mobility or activity.
☐ Yes but only if causing significant pain or discomfort
☐ Yes but only if medical therapy failed
☐ Yes but only if remainder of disease is quiescent.
☐ No there is no role for surgical excision.
☐ Other

State 'other' role for surgical excision of JDM associated calcinosis:

What immunomodulating medications have you used to specifically treat calcinosis?

please note, a question regarding medications that alter calcium and/or phosphate metabolism will follow. This question pertains to any medication that suppresses or alters immune system function
(SELECT ALL THAT APPLY)

☐ Colchicine
☐ Cyclosporine
☐ Methotrexate (if used specifically for calcinosis)
☐ Tacrolimus
☐ Mycophenolate
☐ IVIG
☐ Thalidomide
☐ Systemic glucocorticoids
☐ Local (topical or injected) glucocorticoids
☐ Rituximab
☐ Abatacept
☐ Tocilizumab
☐ Anti-TNF drugs
☐ Other
☐ None

State 'other' immunomodulating medications you have used specifically to treat JDM associated calcinosis:

Rank the top five immunomodulating medications (in order of most successful) you have used when treating JDM associated calcinosis.
Please list only your personal experience
****Select only one drug per ranking position, but mark 'no experience' if applicable****

	First	Second	Third	Fourth	Fifth	No experience
Colchicine	○	○	○	○	○	○
Cyclosporine	○	○	○	○	○	○
Methotrexate (if used specifically for calcinosis)	○	○	○	○	○	○
Tacrolimus	○	○	○	○	○	○
Mycophenolate	○	○	○	○	○	○
IVIG	○	○	○	○	○	○
Thalidomide	○	○	○	○	○	○
Systemic glucocorticoids	○	○	○	○	○	○
Local (topical or injected) glucocorticoids	○	○	○	○	○	○
Rituximab	○	○	○	○	○	○
Abatacept	○	○	○	○	○	○
Tocilizumab	○	○	○	○	○	○
Anti-TNF	○	○	○	○	○	○

Which of the following drugs that alter calcium and/or phosphorous metabolism have you used to specifically treat calcinosis?
(SELECT ALL THAT APPLY)

☐ Sodium thiosulfate IV
☐ Sodium thiosulfate Topical
☐ Bisphosphonates
☐ Probenecid
☐ Diltiazem (or other calcium channel blocker)
☐ Aluminum hydroxide
☐ Other
☐ None

State 'other' drugs which that alter calcium and/or phosphate metabolism that you have used to treat JDM associated calcinosis:

Rank the top three drugs (in order of most successful) which alter calcium and/or phosphorous metabolism that you have used to specifically treat calcinosis.
Please list only your personal experience
****Select only one drug per ranking position, but mark 'no experience' if applicable****

	First	Second	Third	No experience
Sodium thiosulfate IV	○	○	○	○
Sodium thiosulfate Topical	○	○	○	○
Bisphosphonates	○	○	○	○
Probenecid	○	○	○	○
Diltiazem (or other calcium channel blocker)	○	○	○	○
Aluminum hydroxide	○	○	○	○

After beginning targeted treatment for calcinosis, how do you assess response to therapy? (SELECT ALL THAT APPLY)

☐ Patient reported symptoms (if initially present).
☐ Physical exam (physician assessment) if able to be examined.
☐ Imaging - state type.
☐ Laboratory studies related to calcium or phosphorous metabolism.
☐ Laboratory studies related to overall disease activity.
☐ Other

Please list the imaging modalities you use to follow treatment response to targeted calcinosis therapy.

____

List 'other' methods of assessing JDM calcinosis response to therapy:
